# Volatile Compounds from Eggs of Three Fruit Fly Drive Aggregation and Oviposition

**DOI:** 10.3390/insects17030266

**Published:** 2026-03-02

**Authors:** Guofu Ao, Qing’e Ji

**Affiliations:** 1 College of Agriculture, Anshun University, Anshun 561000, China; agf1025@163.com; 2 Key Laboratory of Characteristic and Efficient Agricultural Plant Protection Informatization in Central Guizhou, Anshun 561000, China; 3 Institute of Biological Control, Fujian Agriculture and Forestry University, Fuzhou 350002, China; 4 Key Laboratory of Biopesticide and Chemical Biology, Ministry of Education, Fuzhou 350002, China; 5 The Joint FAO-IAEA Division Cooperation Center for Fruit Fly Control in China, Fuzhou 350002, China

**Keywords:** *Bactrocera dorsalis*, *Zeugodacus cucurbitae*, *Zeugodacus tau*, aggregation, oviposition, volatile compounds

## Abstract

Insects use oviposition secretions containing deterrent signals to regulate intra- and interspecific competition and structure resource partitioning; certain Tephritidae display a striking reversal of this strategy. Herein, we induced female aggregation and oviposition using eggs from the three fruit fly species (*B. dorsalis*, *Z. cucurbitae*, *Z. tau*) and characterized the eggs’ volatile profiles by GC–MS. Within 6 h, female attraction rates to egg stimuli varied significantly by species combination. *B. dorsalis* females were attracted to conspecific eggs at 39.33%, to *Z. cucurbitae* eggs at 28.67%, and to *Z. tau* eggs at 0%. *Z. cucurbitae* females showed attraction rates of 22.67% to *B. dorsalis* eggs, 13.00% to conspecific eggs, and 1.33% to *Z. tau* eggs. *Z. tau* females exhibited 27.67% attraction to *B. dorsalis* eggs, 13.67% to *Z. cucurbitae* eggs, and 18.33% to conspecific eggs. Oviposition assays confirmed strong interspecific effects, with *B. dorsalis* eggs stimulating the greatest egg-laying. GC–MS analysis revealed distinct volatile profiles, with *B. dorsalis* eggs producing the highest number of unique compounds (57), potentially explaining their strong behavioral effects. In total, 79 volatiles differed significantly between *Z. cucurbitae* and *B. dorsalis* eggs, 73 between *Z. tau* and *B. dorsalis* eggs, and 91 between *Z. cucurbitae* and *Z. tau* eggs. These findings reveal a behavioral hierarchy where *B. dorsalis* is the most responsive to egg volatiles, *Z. cucurbitae* is intermediate, and *Z. tau* is the least responsive, a ranking that correlates with significant differences in the eggs’ volatile compositions. This study directly links a behavioral status in interspecific oviposition to species-specific egg volatile profiles.

## Simple Summary

Insect oviposition marks typically deter competitors via signaling compounds that structure resource utilization, yet certain Tephritidae exhibit reversed chemical communication. Females of *Bactrocera dorsalis*, *Zeugodacus cucurbitae*, and *Zeugodacus tau* are attracted to conspecific oviposition cues, resulting in aggregated egg-laying rather than resource partitioning. Using gas chromatography–mass spectrometry (GC–MS) to analyze volatiles from conspecific eggs, we identified species-specific attractive profiles. *B. dorsalis* females showed the strongest aggregation responses, correlating with distinct volatile signatures. These findings confirm that these species lack oviposition-deterring pheromones, instead utilizing attractive semiochemicals to facilitate aggregation.

## 1. Introduction

Insect secretions form protective coatings for egg adhesion and site modification before and after oviposition [1]. Insect oviposition secretions are chemically complex, comprising water, proteins, free amino acids, along with signaling compounds such as oviposition marker pheromones, aggregation pheromones, and altruins [2,3,4,5,6,7,8]. The chemical composition of these secretions mediates host selection and resource utilization among both conspecific and heterospecific insects [9]. Importantly, these chemical signals can also be exploited by natural enemies for host location. For example, the oviposition marker pheromones deposited by *Rhagoletis pomonella* females are used by the parasitoid wasp *Opius lectus* to find hosts and stimulate its own oviposition [10], and egg secretion of *Diprion pini* L. attracts *Chrysonotomyia ruforum Krausse* [11]. Furthermore, females of *R. juglandis*, *R. mendax*, and *B. oleae* deposit oviposition-marking pheromones that deter oviposition by other conspecific females [12,13,14].

The egg-laying behavior of *Bactrocera dorsalis* in mature and near-mature fruits threatens production, as the fruit serves as the primary nutritional resource for hatching larvae. Recent studies have found that *B. dorsalis* eggs and larvae are clustered on the fruits of *Psidium guajava*, and *Mangifera indica* [15,16]. *B. dorsalis* females tend to oviposit repeatedly in host fruits [17], even in the first egg hole they make when laying eggs at low numbers, indicating that *B. dorsalis* do not secrete oviposition marker pheromones during ovipositing. We found in previous field investigations that *Zeugodacus tau* females aggregate to oviposit in cracks, mechanical wounds, or detached stems of old pumpkins. In laboratory assays, we observed that females of *B. dorsalis*, *Z. tau*, and *Z. cucurbitae* aggregated during the oviposition period, depositing eggs on peripheral surfaces such as the edges of rearing cages and the tops of racks. This oviposition on neutral, non-host substrates rather than on or near existing eggs provides behavioral evidence that these species do not secrete host-marking or oviposition-deterring pheromones. The role of egg-derived cues in mediating female aggregation and oviposition remains unexplored in these three species. Here, we analyzed a hexane extract of *B. dorsalis* eggs via GC–MS, identifying 12 compounds including myristic alcohol, ethyl myristate, methyl laurate, and ethyl laurate. These compounds have previously been shown to elicit consistent antennal (EAG) and behavioral responses in the parasitoid *Fopius arisanus* [18]. Analysis of egg surface extracts identified 11 compounds from *B. dorsalis* and 7 from *B. correcta*. Four of these, including anethole, dodecanoic acid, dodecanoic acid ethyl ester, and (Z)-11-tetradecenoic acid, were unique to *B. dorsalis* eggs [19]. However, the volatile profiles of *Z. tau* and *Z. cucurbitae* eggs remain uncharacterized, and a comparative analysis of volatiles across *B. dorsalis*, *Z. cucurbitae*, and *Z. tau* has not been conducted.

This study investigated the role of eggs from *B. dorsalis*, *Z. cucurbitae*, and *Z. tau* in mediating aggregation and oviposition, both within and among species. Volatile compounds emitted by eggs from all three species were identified using headspace solid-phase microextraction (SPME) coupled with GC–MS. These findings provide a foundation for elucidating the specific chemical cues that trigger female aggregation and oviposition.

## 2. Materials and Methods

### 2.1. Insect Collection and Rearing

*B. dorsalis* females were collected from infested mango trees in Fuzhou City, Fujian Province, China, and subsequently reared in the laboratory for 15 generations. *Z. cucurbitae* and *Z. tau* colonies, maintained in the laboratory for over 30 generations, were used for the experiments. Insects were reared at the Institute of Biological Control, Fujian Agriculture and Forestry University. Adults were maintained in cages of two sizes (25 cm × 25 cm × 25 cm or 62 cm × 99 cm × 116 cm) with ad libitum access to food and water under controlled conditions of 25 ± 2 °C, 65 ± 5% relative humidity (RH), and a 12L:12D photoperiod [20].

### 2.2. Egg Collection and Female Attraction

Laboratory-reared *B. dorsalis* adults were maintained in large cages for 10–20 days. For egg collection at designated time points, egg-collecting bottles were first misted with 4–5 mL of sterile water and then placed inside the rearing cage [21]. Adults of *Z. tau* and *Z. cucurbitae* were maintained under similar conditions for 15–25 days following laboratory adaptation. Using the same protocol, 4–5 mL of sterile water was added to egg-collecting bottles, which were then swirled to coat the inner walls; excess water was discarded. Subsequently, 20–25 g portions of pumpkin were weighed, placed into the bottles, and used to collect eggs at specific intervals.

#### 2.2.1. Fruit Fly Trap

The trap consisted of a flat-bottom glass tube (4 cm diameter × 13 cm height). Fruit fly eggs were placed at the bottom, and two white filter papers (6 cm × 6 cm) were positioned above them. Sterile water (600 µL) was added to moisten the filter papers, and the tube opening was sealed with plastic film. A transparent plastic tube (0.8 cm diameter × 3 cm height) was inserted through the center of the sealing film; the interior and exterior surfaces of this tube were coated with wet adhesive powder to a height of approximately 0.5 cm from the base. A control containing an equivalent volume of sterile water in place of eggs was prepared identically. Finally, the entire glass tube was wrapped in white paper to standardize visual cues [21].

#### 2.2.2. Oviposition Container

The container used to attract females to oviposit was a flat-bottom glass tube (Φ 2.5 cm, h 9 cm). A fruit egg was placed at the bottom of the glass tube. Two white filter papers (3 cm × 3 cm), each with two central holes, were suspended 2.0–2.5 cm above the attractant at the bottle mouth. Sterile water (600 μL) was added to the system, and the glass tube was wrapped with white paper [21].

#### 2.2.3. Test Cage

Behavioral trials used a 30 cm^3^ 100-mesh cage mounted on a white plastic plate fitted with a rotating base (1–2 rpm). Adult feed was placed in the center of the bottom of the cage, and a white sponge containing water was placed at the top [21]. Details regarding the experimental conditions, insect rearing, and egg collection are presented in the Supplementary File S1.

### 2.3. Influence of B. dorsalis, Z. cucurbitae, and Z. tau Eggs on Aggregation and Oviposition Behaviors

Fifty females of each species *B. dorsalis* [15 days old], *Z. cucurbitae* [15 days old], and *Z. tau* [20 days old] were released into test cages. *B. dorsalis* eggs (0.8 g) were weighed and divided equally between two traps (or oviposition containers) positioned on the left and right sides of the cage, 2 cm from the edges. The number of females attracted and those that oviposited were recorded over 6 h. Control cages received an equivalent volume (0.8 g) of sterile water. This procedure was replicated six times. Subsequently, pumpkin seeds and eggs of *Z. tau* and *Z. cucurbitae* (0.8 g each) were tested using the same methodology. The trap (or oviposition container) consisted of an equilateral triangle with 26 cm sides. Female attraction and oviposition were monitored for 6 h, with sterile water serving as the control. All assays comprised six replicates.

Attraction rate (%) = number of adults captured/total number of adults tested × 100

### 2.4. GC–MS Analysis of Volatile Compounds from B. dorsalis, Z. cucurbitae, and Z. tau Eggs

For volatile analysis, 2.0 g samples of eggs or pumpkin were flash-frozen in liquid nitrogen (30–60 s) in cryovials. Then, 500 mg aliquots were transferred to headspace vials and spiked with 10 µL of 2-octanol (internal standard). The volatile compounds in the samples were analyzed by GC–MS. The analysis was performed five times.

In situ solid-phase microextraction (ISPME) was performed using a PAL rail system under the following conditions: incubation at 60 °C for 30 min following a 15 min preheat, and desorption for 4 min. GC–MS analysis was conducted on an Agilent (Santa Clara, CA, USA) 7890 gas chromatograph coupled to a 5977B mass spectrometer (Santa Clara, CA, USA) equipped with a DB-WAX column in splitless mode. Helium carrier gas was set at 1 mL min^−1^ with a front inlet purge flow of 3 mL min^−1^. The oven temperature program started at 40 °C (held 4 min), ramped to 245 °C at 5 °C min^−1^, and held for 5 min. The injection port, transfer line, ion source, and quadrupole temperatures were 250, 250, 230, and 150 °C, respectively. Electron ionization energy was 70 eV. Mass spectra were acquired in scan mode over the m/z range 20–400 with no solvent delay.

### 2.5. Data Processing and Analysis

The number of females attracted to the traps was recorded. Eggs from the oviposition containers were collected, placed on black filter paper, and photographed using a Nikon D750 camera (Nikon Corporation, Tokyo, Japan) in macro mode. ImageJ software (v1.8.0) was used for image processing and egg counting. For attraction rate data, differences among the three attractants (Control, Pumpkin, and Treatments) were analyzed using one-way analysis of variance (ANOVA), followed by Tukey’s Honestly Significant Difference (HSD) post hoc test for pairwise comparisons. For egg count data of *B. dorsalis*, differences between Control and Treatments were analyzed using Student’s *t*-test (for normally distributed data) or Mann–Whitney U test (for non-normally distributed data). Normality was assessed using the Shapiro–Wilk test prior to parametric analysis. All statistical analyses were performed using SPSS v.22.0.

Data processing was performed in ChromaTOF 4.3X (LECO Corporation, St. Joseph, MI, USA), where raw peaks were extracted, baseline-corrected, aligned, and deconvoluted. Peak areas were then integrated and matched against the NIST spectral library [22]. After noise filtering (single-peak removal) and median imputation of missing values, data were normalized to the internal standard. Subsequent preprocessing—log transformation, centering, and UV scaling—was performed using SIMCA (v.16.0.2, Sartorius Stedim Data Analytics AB, Umeå, Sweden) [23]. The card value standard used was a *p*-value < 0.05 on Student’s *t*-test, and the variable importance in the projection of the first principal component of the orthogonal projections to latent structures discriminant analysis was greater than 1. Differential volatiles were screened and analyzed. The GC–MS metabolomics data were analyzed by Shanghai Baiqu Biomedical Technology Co., Ltd. (Shanghai, China) The volcano plots, we have added that these were generated in R version 4.3.1 using the Enhanced Volcano package (v1.18.0).

## 3. Results

### 3.1. Inducing Fruit Fly Females to Aggregate Using B. dorsalis, Z. cucurbitae, and Z. tau Eggs

**Figure 1:**
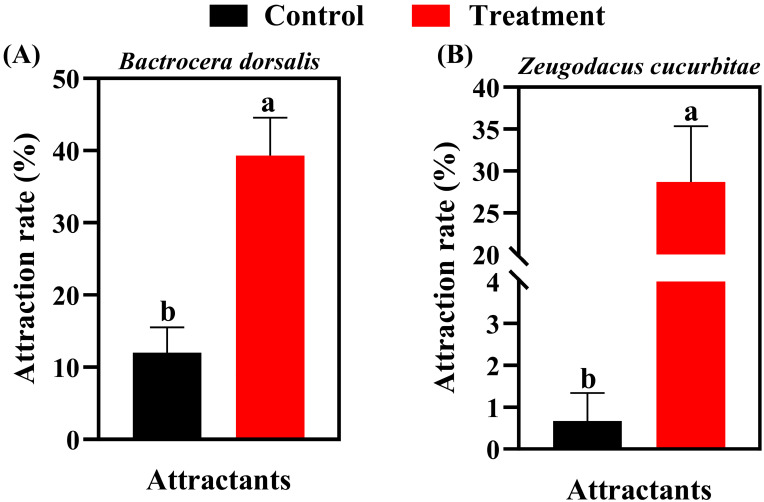
Number of attracted females observed in response to *B. dorsalis* eggs. Panels show responses from (**A**) *Bactrocera dorsalis* and (**B**) *Zeugodacus cucurbitae* females. Data are expressed as mean ± standard error (*n* = 6). Different lowercase letters denote significant differences at *p* < 0.05.

**Figure 2:**
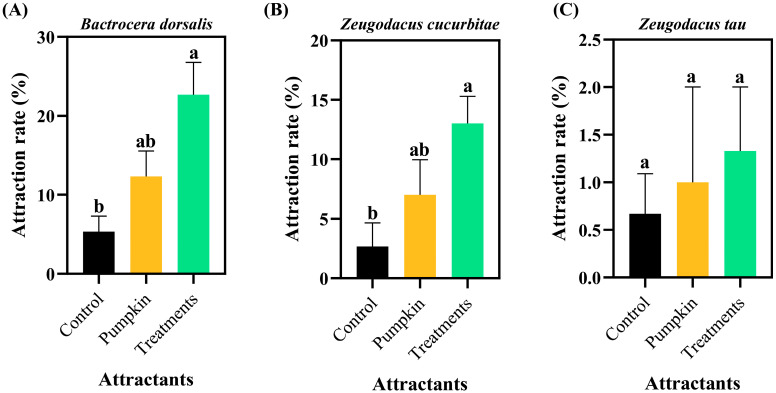
Attraction responses of females from three species to *Z. cucurbitae* eggs. (**A**) *Bactrocera dorsalis*; (**B**) *Zeugodacus cucurbitae*; (**C**) *Zeugodacus tau*. Data are expressed as the mean ± standard error. Lowercase letters indicate significant differences at *p* < 0.05.

**Figure 3:**
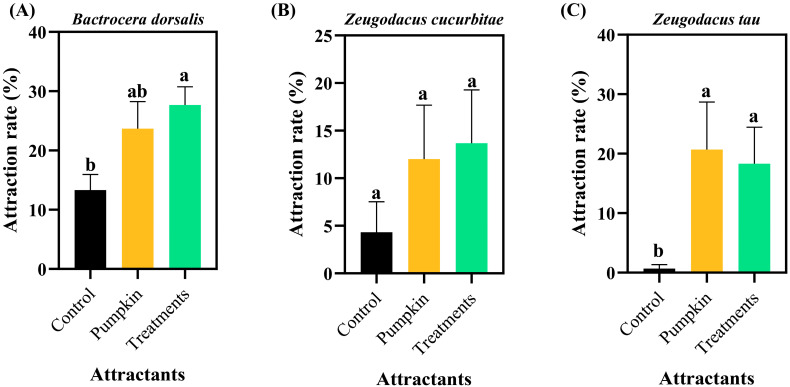
Attraction responses of females from three species to *Z. tau* eggs. (**A**) *Bactrocera dorsalis*; (**B**) *Zeugodacus cucurbitae*; (**C**) *Zeugodacus tau*. Data are expressed as the mean ± standard error. Lowercase letters indicate significant differences at *p* < 0.05.

*B. dorsalis* eggs showed significant attraction to conspecific and *Z. cucurbitae* females within 6 h (39.33% and 28.67%, respectively; Figure 1A), with both rates being significantly higher than the control (*p* < 0.05). No *Z. tau* females were attracted to *B. dorsalis* eggs within 6 h, and this result did not differ significantly from the control. Within 6 h, *Z. cucurbitae* eggs attracted 22.67% of *B. dorsalis* females (Figure 2A) and 13.00% of conspecific females (Figure 2B). Statistically, these values were not significantly different from the pumpkin control but were significantly higher than the standard control (*F*_2,15_ = 5.674, *p* = 0.015 and *F*_2,15_ = 4.826, *p* = 0.024, respectively). The attraction rate of *Z. cucurbitae* eggs to *Z. tau* females within 6 h was 1.33% (Figure 2C), which did not differ significantly from that of pumpkin or the control (*F*_2,15_ = 0.205, *p* = 0.817). *Z. tau* eggs attracted 27.67% of B. dorsalis females within 6 h (Figure 3A). This rate was not significantly different from the pumpkin control but was significantly higher than the standard control (*F*_2,15_ = 4.412, *p* = 0.031). The attraction rate of *Z. tau* eggs to *Z. cucurbitae* females within 6 h was 13.67% (Figure 3B), which did not differ significantly from that of pumpkin or the control (*F*_2,15_ = 1.003, *p* = 0.390). The attraction rate of *Z. tau* eggs to their females within 6 h was 18.33% (Figure 3C), which did not differ significantly from that of the pumpkin but was significantly higher than that of the control (*F*_2,15_ = 3.515, *p* = 0.049).

### 3.2. Effects of B. dorsalis, Z. cucurbitae, and Z. tau Eggs on Oviposition of Females

**Figure 4:**
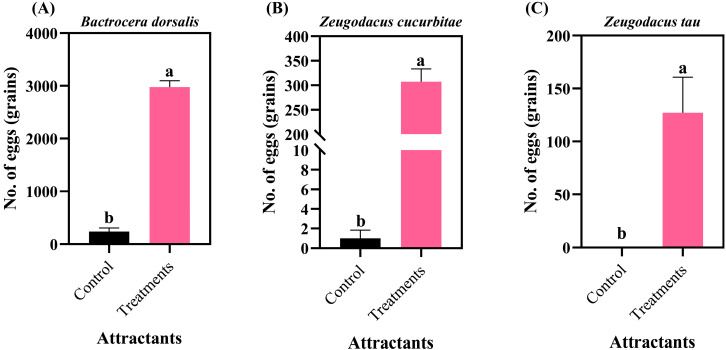
Oviposition responses of three fruit fly species to *B. dorsalis* eggs. (**A**) *Bactrocera dorsalis*; (**B**) *Zeugodacus cucurbitae*; (**C**) *Zeugodacus tau*. Data are expressed as the mean ± standard error. Lowercase letters indicate significant differences at *p* < 0.05.

*B. dorsalis* eggs induced conspecific females to produce 2973.50 eggs within 6 h (Figure 4A), which was significantly higher than the control (*p* < 0.05). When tested on heterospecific females, *B. dorsalis* eggs induced *Z. cucurbitae* females to produce 307.33 eggs (Figure 4B), significantly higher than controls (*p* < 0.05), and *Z. tau* females to produce 127.00 eggs (Figure 4C), significantly higher than controls (*p* < 0.05).

**Figure 5:**
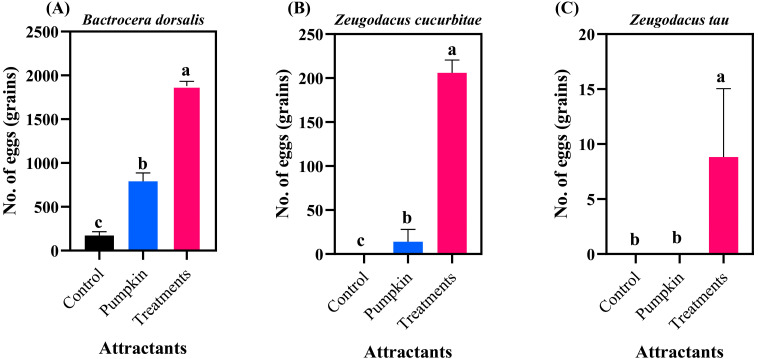
Oviposition responses of three fruit fly species to *Z. cucurbitae* eggs. (**A**) *Bactrocera dorsalis*; (**B**) *Zeugodacus cucurbitae*; (**C**) *Zeugodacus tau*. Data are expressed as the mean ± standard error. Lowercase letters indicate significant differences at *p* < 0.05.

*Z. cucurbitae* eggs elicited high oviposition in heterospecific *B. dorsalis* females (1878.17 eggs; Figure 5A), significantly exceeding controls (*F*_2,15_ = 159.734, *p* < 0.0001). They also stimulated conspecific females to lay 206.00 eggs (Figure 5B), significantly higher than controls (*F*_2,15_ = 98.516, *p* < 0.0001). However, *Z. cucurbitae* eggs induced only 8.83 eggs from heterospecific *Z. tau* females (Figure 5C), with no significant difference from pumpkin or control treatments (*F*_2,15_ = 2.023, *p* = 0.167).

**Figure 6:**
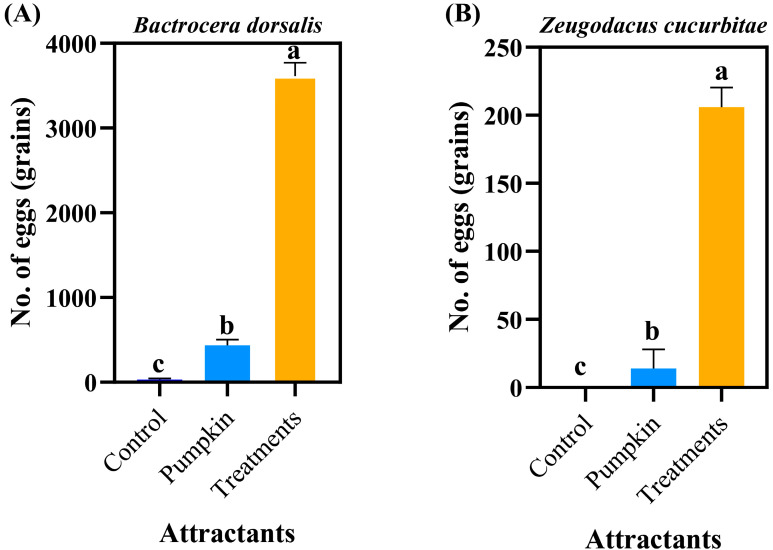
Oviposition responses of three fruit fly species to *Z. tau* eggs. (**A**) *Bactrocera dorsalis*; (**B**) *Zeugodacus cucurbitae*. Data are expressed as the mean ± standard error. Lowercase letters indicate significant differences at *p* < 0.05.

*Z. tau* eggs elicited strong oviposition responses from heterospecific females, inducing *B. dorsalis* females to lay 3613.83 eggs and *Z. cucurbitae* females to lay 295.67 eggs within 6 h (Figure 6A,B). Both totals were significantly higher than those produced in response to pumpkin and standard controls (*B. dorsalis*: *F*_2,15_ = 394.935, *p* < 0.0001; *Z. cucurbitae*: *F*_2,15_ = 44.686, *p* < 0.0001). In contrast, conspecific *Z. tau* females did not oviposit in response to *Z. tau* eggs, pumpkin, or control treatments, with no significant difference observed among these three groups.

### 3.3. Identification of Volatile Compounds from B. dorsalis, Z. cucurbitae, and Z. tau Eggs

**Figure 7:**
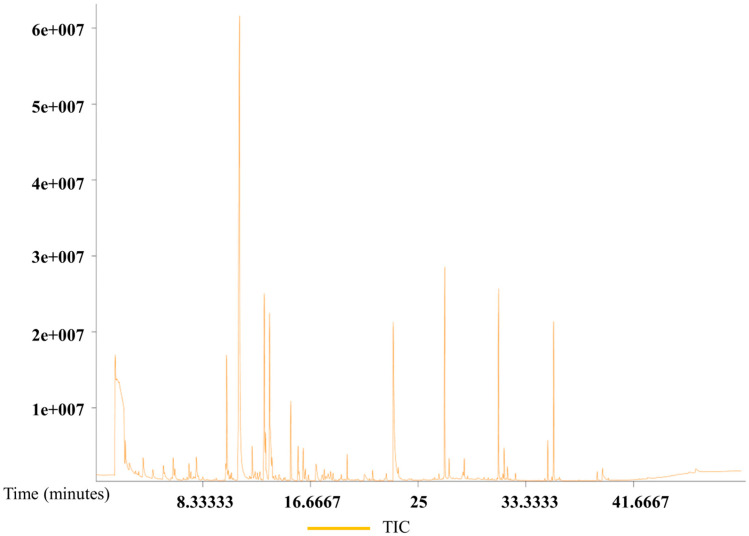
Gas chromatography–mass spectrometry total ion chromatogram of *B. dorsalis* eggs.

**Figure 8:**
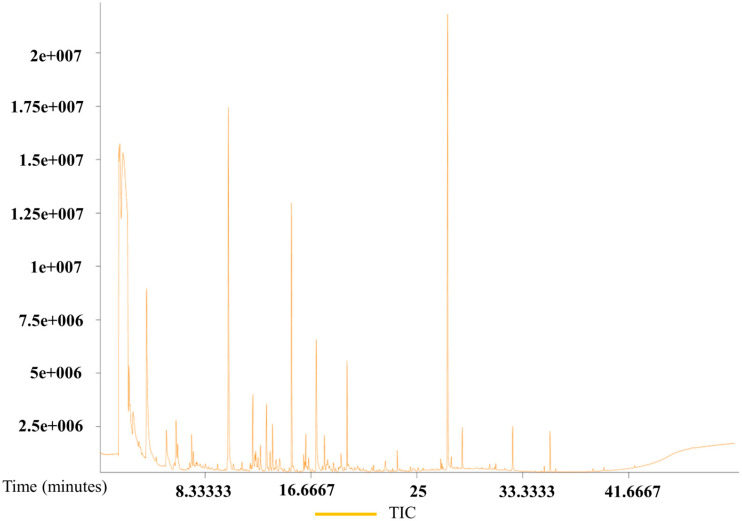
Gas chromatography–mass spectrometry total ion chromatogram of *Z. cucurbitae* eggs.

**Figure 9:**
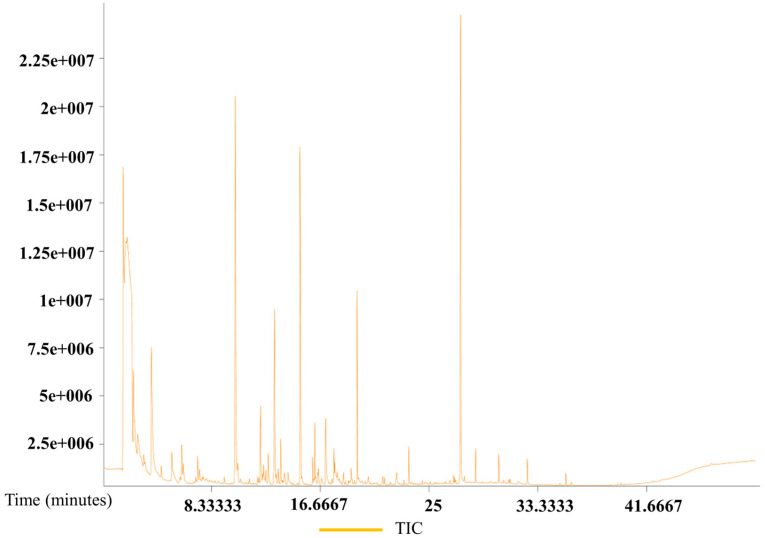
Gas chromatography–mass spectrometry total ion chromatogram of *Z. tau* eggs.

The total ion current chromatograms from the GC–MS analysis of eggs from all three species are presented in Figure 7, Figure 8 and Figure 9. The chromatograms demonstrate good sample quality, system stability, and a stable baseline, indicating reliable analytical conditions.

GC–MS analysis identified a total of 159 volatile compounds (similarity > 500) from the eggs of all three species and pumpkin (Table S1). Among these, *B. dorsalis* eggs alone contained 131 volatile compounds, of which 22 were also present in pumpkin. The 131 volatile compounds from *B. dorsalis* eggs were categorized as follows: 41 esters (79.80% relative content), 21 alkanes (3.04%), 17 other compounds (2.76%), 8 acids (2.01%), 10 ketones (7.88%), 5 amines (1.12%), 10 alcohols (0.77%), 1 olefin (0.04%), and 18 unknown compounds (1.00%). The 87 volatiles identified from *Z. cucurbitae* eggs included 19 shared with pumpkin and were categorized as 14 esters (32.48%), 22 alkanes (19.78%), 9 alcohols (12.44%), 6 amines (9.99%), 8 ketones (8.41%), 11 other compounds (7.47%), 12 unknown compounds (6.79%), 3 alkenes (2.24%), and 2 acids (0.36%). Analysis of *Z. tau* eggs identified 93 volatile compounds, including 20 shared with pumpkin. The major classes were 17 esters (26.29%), 22 alkanes (18.03%), 8 ketones (16.45%), and 12 other compounds (13.15%), with the remainder consisting of alcohols, amines, unknown compounds, acids, and olefins. Comparative analysis revealed a shared volatile signature of 58 compounds across the three species, 8 of which are currently uncharacterized. *B. dorsalis* and *Z. cucurbitae* shared 5 common compounds, including 1 unknown compound, whereas 11 compounds were the same between *B. dorsalis* and *Z. tau*, and 22 between *Z. cucurbitae* and *Z. tau*. Fifty-seven compounds were unique to *B. dorsalis*, including nine unknown compounds. Four compounds were found to be specific to each of *Z. cucurbitae* and *Z. tau*.

### 3.4. Analysis of Different Volatile Compounds in B. dorsalis, Z. cucurbitae, and Z. tau Eggs

**Figure 10:**
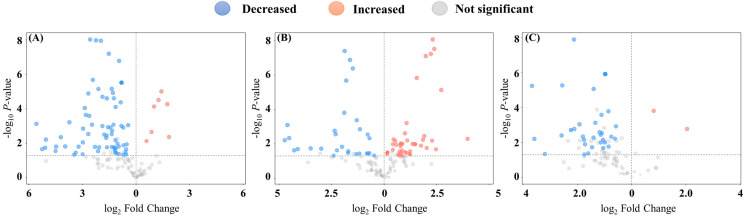
Differential metabolite volcanic plot for the three fruit fly species. (**A**) *B. dorsalis* and *Z. cucurbitae* groups; (**B**) *B. dorsalis* and *Z. tau* groups; (**C**) *Z. cucurbitae* and *Z. tau* groups.

A volcano plot was generated to statistically analyze and visualize significant differences in the abundance of volatile compounds between sample groups. Statistical analysis of 159 different volatiles revealed 79 significantly different volatiles between *Z. cucurbitae* and *B. dorsalis*, with 7 increased, including 1,4-pentadiene, 4-penten-2-ol, 3-butyl-2-hydroxy-2-cyclopenten-1-one, 2-[2-(2-acetyloxyethoxy)phenoxy]ethyl acetate, 1,4-dichlorobenzene, 1,4,6-trimethyl-2(1H)-pyridinone, and formamide. In contrast, 72 volatiles were significantly decreased. These included compounds such as ethyl 9-tetradecenoate, isoamyl laurate, myristic acid, lauric acid, methyl tetradecanoate, 6-methylheptan-2-one, and 5-hydroxy-2,7-dimethyloctan-4-one (Figure 10A). Between *Z. tau* and *B. dorsalis*, 73 volatiles differed significantly, with 44 volatiles increased, including 1,4-pentadiene, 4-penten-2-ol, methyl acetate, isobutyl 2-methylbutyrate, cyclopentanone, 2-nonanol, and (*Z*)-14-tricosenyl formate. A set of 29 volatiles, including 6-methylheptan-2-one, anethole, and isoamyl laurate, was decreased (Figure 10B). Between *Z. cucurbitae* and *Z. tau*, 91 volatiles differed significantly. N-Hexyl-propanamide and 1-hydrocyclohexanecarboxylic acid were enhanced, while 89 volatiles, including 6-ethyl-2-methyloctane, 6-methylheptan-2-one, ethyl 9-tetradecenoate, isoamyl laurate, (*Z*)-14-tricosenyl formate, 2-tridecanone, and isobutyl laurate, were reduced (Figure 10C).

## 4. Discussion

The selection of suitable oviposition sites is critical for reproductive success, as host quality directly determines larval developmental fitness and long-term population persistence. Suitable hosts must satisfy nutritional requirements for offspring while also facilitating adult behaviors such as mating and sheltering. Tephritid females commonly oviposit into fruit, assessing sites based on host species, ripeness, location, color, shape, and volatile cues [24,25,26,27,28,29]. Oviposition decisions are further influenced by the insect’s own genetic background, physiological condition, and habitat structure [30], alongside competitive interactions that may be intraspecific or interspecific [14]. Host-derived nutrients and secondary metabolites consequently mediate insect growth, fecundity, and population stability [31]. Beyond the known attraction to mature fruit volatiles, we found that the eggs of *B. dorsalis*, *Z. cucurbitae*, and *Z. tau* also emit attractants, inducing oviposition in both conspecific and heterospecific females. Specifically, eggs from all three species significantly stimulated oviposition in *B. dorsalis* and *Z. cucurbitae* females compared to the pumpkin and standard controls (Figure 1, Figure 2, Figure 3, Figure 4 and Figure 5). Additionally, *B. dorsalis* eggs alone significantly increased oviposition rates in *Z. tau* females relative to the control. Modern insect taxonomy integrates data from morphology, genetics, molecular biology, physiology, and chemical ecology to delineate new species and determine their closest phylogenetic relatives. *Culex quinquefasciatus* and *C. pipiens* eggs secretes pheromones with the same active components, i.e., 1,3-diacylglycerol, dodecyl carbonate, and tetradecyl carbonate but differed from those of *C. tarsalis* eggs [32]. The components of *B. dorsalis*, *Z. cucurbitae*, and *Z. tau* eggs were analyzed by GC–MS, which identified 131, 87, and 93 compounds, respectively (Table S1). Of these, 58 compounds were common in all three fruit fly eggs; 5 between *B. dorsalis* and *Z. cucurbitae*; 11 between *B. dorsalis* and *Z. tau*; and 22 between *Z. cucurbitae* and *Z. tau*. Moreover, 57 compounds were unique to *B. dorsalis*, whereas 4 were unique to each of *Z. cucurbitae* and *Z. tau* (Table S1).

The compounds found as attractants for fruit flies include esters, ketones, amines, and alcohols [33,34,35], while esters, ketones, alkanes, and acids were found in the aggregation pheromone released in the oviposition secretions of insects, such as *Schistocerca gregaria*, *Culex quinquefasciatus*, *Aedes aegypti*, and *Simulium damnosum* [6,36,37]. Therefore, future work to identify the active aggregation and oviposition compounds in these fruit flies will focus on screening esters and ketones.

Furthermore, this study found that the volatile substances in the fruits of some plants were the same as those in the eggs of *B*. *dorsalis*, *Z. cucurbitae*, and *Z. tau* (Table S1), suggesting that fruit flies use these substances for growth, development, and reproduction. For example, myristic and palmitoleic acids in *Actinidia chinensis* fruit [38]; isoamyl isovalerate and isobutyl isovalerate in mature *Musa nana* fruit [39]; and anethole in green ripe *Averrhoa carambola* [33] were detected in *B*. *dorsalis*, *Z. cucurbitae*, and *Z. tau* eggs.

## 5. Conclusions

This study reveals that *B. dorsalis*, *Z. cucurbitae*, and *Z. tau* utilize egg-derived volatiles as aggregation cues, representing a strategy distinct from the oviposition-deterring pheromones typical of other tephritids. The identified behavioral hierarchy, coupled with species-specific volatile profiles, offers practical opportunities for developing targeted monitoring systems and oviposition attractants. The complex volatile blend of *B. dorsalis* eggs, in particular, represents a promising lead for synthetic lure development. Future work should prioritize the isolation and bioassay of individual compounds within these blends, assess efficacy in field cage trials, and explore the potential for these cues to enhance existing biological control strategies through improved detection and population monitoring.

## Data Availability

The original contributions presented in this study are included in the article/Supplementary Materials. Further inquiries can be directed to the corresponding author.

## Supplementary Materials

The following supporting information can be downloaded at: https://www.mdpi.com/article/10.3390/insects17030266/s1, Table S1: Volatile compounds and relative contents in eggs of *Bactrocera dorsalis*, *Zeugodacus cucurbitae*, and *Zeugodacus tau* (%). File S1: Eggs collection methodology.

## References

[1] Lu J.B., Ren P.P., Tian Y., Yang Y.Y., Feng Q.K., Zhang X.Y., He F., Huang H.J., Chen J.P., Li J.M. (2025). Structural characterization and proteomic profiling of oviposition secretions across three rice planthopper species. Insect Biochem. Mol. Biol..

[2] Mudd A., Ferguson A.W., Blight M.M., Williams I.H., Scubla P., Solinas M., Clark S.J. (1997). Extraction, isolation, and composition of oviposition-deterring secretion of cabbage seed weevil *Ceutorhynchus assimilis*. J. Chem. Ecol..

[3] Yang L.L., Gao Q.P., Dai J.J., Yuan G.Z., Wang L., Qian C., Zhu B.J., Liu C.L., Wei G.Q. (2018). Comparative transcriptome analysis of silkworm, *Bombyx mori* colleterial gland suggests their functional role in mucous secretion. PLoS ONE.

[4] Pervez A., Gupta A.K. (2004). Role of surface chemicals in egg cannibalism and intraguild predation by neonates of two aphidophagous ladybirds, *Propylea dissecta* and *Coccinella transversalis*. J. Appl. Entomol..

[5] Oliver T.H., Timms J.E.L., Taylor A., Leather S.R. (2006). Oviposition responses to patch quality in the larch ladybird *Aphidecta obliterata* (Coleoptera: Coccinellidae): Effects of aphid density, and con-and heterospecific tracks. B. Entomol. Res..

[6] Coupland J.B. (1991). Oviposition response of *Simulium reptans* (Diptera: Simuliidae) to the presence of conspecific eggs. Ecol. Entomol..

[7] Suiter D.R., Carlson D.A., Patterson R.S., Koehler P.G. (1996). Host-location kairomone from *Periplaneta americana* (L.) for parasitoid *Aprostocetus hagenowii* (Ratzeburg). J. Chem. Ecol..

[8] Schröder R., Cristescu S.M., Harren F.J.M., Hilker M. (2007). Reduction of ethylene emission from *Scots pine* elicited by insect egg secretion. J. Exp. Bot..

[9] Zhang X., Wang G. (2025). Sources, identification, and behavioral significance of oviposition-deterring pheromones in insects. Pest Manag. Sci..

[10] Prokopy R.J., Webster R.P. (1978). Oviposition-deterring pheromone of *Rhagoletis pomonella*. J. Chem. Ecol..

[11] Hilker M., Stein C., Schröder R., Varama M., Mumm R. (2005). Insect egg deposition induces defence responses in *Pinus sylvestris*: Characterisation of the elicitor. J. Exp. Biol..

[12] Girolami V., Vianello A., Strapazzon A., Ragazzi E., Veronese G. (1981). Ovipositional deterrents in *Dacus oleae*. Entomol. Exp. Appl..

[13] Stelinski L.L., Rodriguez-Saona C., Meyer W.L. (2009). Recognition of foreign oviposition-marking pheromone in a multi-trophic context. Naturwissenschaften.

[14] Nufio C.R., Papaj D.R. (2004). Host-marking behaviour as a quantitative signal of competition in the walnut fly *Rhagoletis juglandis*. Ecol. Entomol..

[15] Zhao J.P., Ma J., Wu M.T., Jiao X.G., Wang Z.G., Liang F., Zhan G.P. (2017). Gamma radiation as a phytosanitary treatment against larvae and pupae of *Bactrocera dorsalis* (Diptera: Tephritidae) in guava fruits. Food Control.

[16] Soemargono A., Muryati M., Hasyim A., Istianto M. (2011). Spatial distribution pattern of the fruit fly, *Bactrocera dorsalis* complex (Diptera: Tephritidae) in mango orchard. Agrivita J. Agric. Sci..

[17] Theron C.D., Kotzé Z., Manrakhan A., Weldon C.W. (2023). Oviposition by the oriental fruit fly, *Bactrocera dorsalis* (Hendel) (Diptera: Tephritidae), on five citrus types in a laboratory. Austral Entomol..

[18] Ji Q.E., Bi K., Chen J.H. (2016). Response of egg-pupal parasitoid *Fopius arisanus* (Sonan) to infochemicals from the host eggs’ surface of *Bactrocera dorsalis* (Hendel). J. Asia-Pac. Entomol..

[19] Wei B., Wei C.M., Li Y.G., Tang J.C., Hu X.S., Liu H., Dong W.X. (2021). The effect of egg extracts on the behavior of gravid female *Bactrocera dorsalis* and *Bactrocera correcta* and analysis of chemicals on the egg surfaces of these species. Chin. J. Appl. Entomol..

[20] Lin J., Hao X.X., Yue G.Q., Yang D.Q., Lu N.F., Cai P.M., Ao G.F., Ji Q.E. (2022). Efficacy of wax-based bait stations for controlling *Bactrocera dorsalis* (Diptera: Tephritidae). Pest Manag. Sci..

[21] Li H.J., Ren L., Xie M.X., Gao Y., He M.Y., Hassan B., Lu Y.Y., Cheng D.F. (2020). Egg-Surface bacteria are indirectly associated with oviposition aversion in *Bactrocera dorsalis*. Curr. Biol..

[22] Kind T., Wohlgemuth G., Lee D.Y., Lu Y., Palazoglu M., Shahbaz S., Fiehn O. (2009). FiehnLib: Mass spectral and retention index libraries for metabolomics based on quadrupole and time-of-flight gas chromatography/mass spectrometry. Anal. Chem..

[23] Wiklund S., Johansson E., Sjöeströem L., Mellerowicz E.J., Edlund U., Shockcor J.P., Johan Gottfries Moritz T., Trygg J. (2008). Visualization of GC/TOF-MS-based metabolomics data for identification of biochemically interesting compounds using OPLS class models. Anal. Chem..

[24] Singh S., Sharma D.R. (2013). Biology and morphometry of *Bactrocera dorsalis* and *Bactrocera zonata* on different fruit crops. Indian J. Agric. Sci..

[25] Dane K.M., Johnson M.W. (2010). Olive fruit fly: Managing an ancient pest in modern times. Annu. Rev. Entomol..

[26] Brévault T., Quilici S. (2009). Oviposition preference in the oligophagous tomato fruit fly, *Neoceratitis cyanescens*. Entomol. Exp. Appl..

[27] Somta C., Winotai A., Ooi P.A.C. (2009). Fruit flies reared from *Terminalia catappa* in Thailand. J. Asia-Pac. Entomol..

[28] Guillén L., Sivinski J., Rull J. (2016). The role of males in host-fruit selection by females of a walnut infesting Tephritid (Diptera) *Rhagoletis zoqui*. J. Insect Behav..

[29] Katsoyannos B.I., Kouloussis N.A. (2001). Captures of the olive fruit fly *Bactrocera oleae* on spheres of different colours. Entomol. Exp. Appl..

[30] Browne L.B. (1993). Physiologically induced changes in resource-oriented behavior. Ann. Rev. Entomol..

[31] Papachristos D.P., Papadopoulos N.T. (2009). Are citrus species favorable hosts for the Mediterranean fruit fly? A demographic perspective. Entomol. Exp. Appl..

[32] Starratt A.N., Osgood C.E. (1973). 1,3-Diglycerides from eggs of *Culex pipiens quinquefascitus* and *Culex pipiens*. Comp. Biochem. Physiol. B: Biochem. Mol. Biol..

[33] Eisemann C.H., Rice M.J. (2011). Attractants for the gravid queensland fruit fly *Dacus tryoni*. Entomol. Exp. Appl..

[34] Kamala Jayanthi P.D., Kempraj V., Aurade R.M., Bruce T.J.A. (2017). Evaluation of synthetic oviposition stimulants to enhance egg collection of the oriental fruit fly, *Bactrocera dorsalis* (Diptera: Tephritidae). J. Pest Sci..

[35] Serra N.S., Garrido C.M., Català A.B., Tait G., Merli D., Carlin S., Malacrida A.R., Gasperi G., Anfora G., Scolari F. (2021). Electrophysiological responses of the Mediterranean fruit fly, *Ceratitis capitata*, to the cera trap lure: Exploring released antennally-active compounds. J. Chem. Ecol..

[36] Braks M.A.H., Leal W.S., Cardé R.T. (2007). Oviposition responses of gravid female *Culex quinquefasciatus* to egg rafts and low doses of oviposition pheromone under semifield conditions. J. Chem. Ecol..

[37] Ganesan K., Mendki M.J., Suryanarayana M.V.S., Prakash S., Malhotra R.C. (2006). Studies of *Aedes aegypti* (Diptera: Culicidae) ovipositional responses to newly identified semiochemicals from conspecific eggs. Aust. J. Entomol..

[38] Young H., Paterson V.J., Burns D.J.W. (2010). Volatile aroma constituents of kiwifruit. J. Sci. Food Agric..

[39] Pino J.A., Ortega A., Marbot R., Aguero J. (2003). Volatile components of banana fruit (*Musa sapientum* L.) “Indio” from Cuba. J. Essent. Oil Res..

