# Supplementary material for: Volatile Compounds from Eggs of Three Fruit Fly Drive Aggregation and Oviposition
Source: Insects. 2026 Mar 2;17(3):266. doi: 10.3390/insects17030266 (PMC13027054; doi:10.3390/insects17030266)

Collecting egg

The large insect rearing cage (62 cm × 99 cm × 116 cm) is a container for rearing adult fruit flies, with egg-laying holes of different quantities on both sides. During the experiment, a large number of fruit fly eggs can be collected after placing the egg-laying bottle into the holes for a certain period of time.

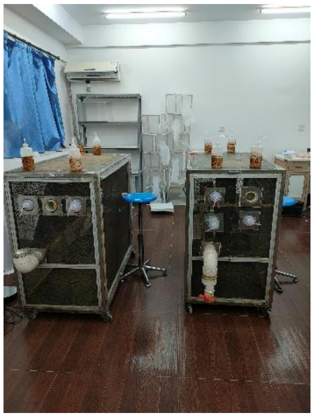

Fabrication of egg collecting device

Self-made egg collecting bottles (Φ7 cm, h 16 cm, V 500 mL) were loaded with two pieces of filter paper (3 cm×6 cm), wrapped with newspaper, and sterilized in a drying oven at 80 °C for 6 h. Then 5 mL of sterile water was added in an ultra-clean workbench, and the bottles were shaken to make the water adhere to the inner walls, followed by pouring off the excess sterile water. The egg-collecting bottles were placed into the egg-collecting holes of large insect rearing cages for egg collection and taken out for standby after 6 h.

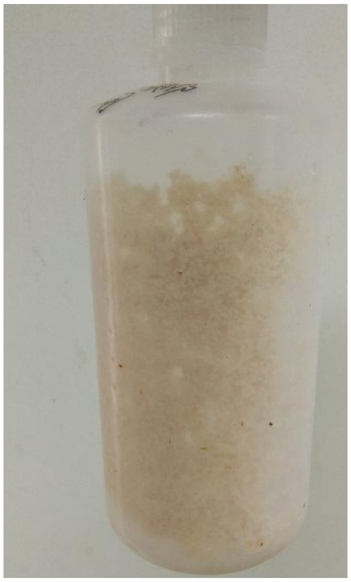

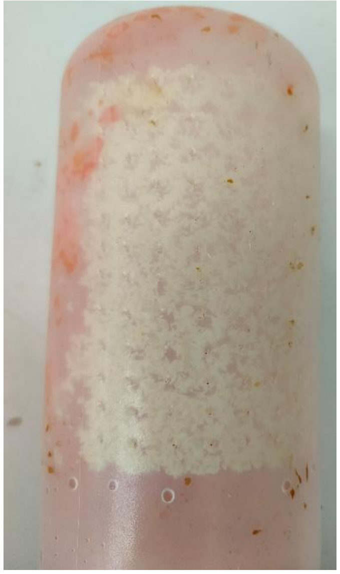

Fabrication of fly trap

A certain amount of eggs was weighed and placed at the bottom of flat-bottom glass tubes (Φ4 cm, h 13 cm). Two pieces of white filter paper (6 cm×6 cm) were placed above the eggs, and 600 μL of sterile water was added. The tube orifices were sealed with parafilm, and a transparent plastic tube (Φ0.8 cm, h 3 cm) was inserted into the center of the parafilm. The glass tubes were wrapped with white paper.

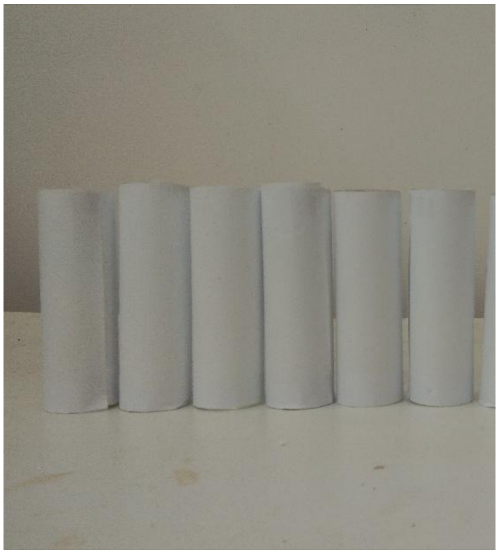

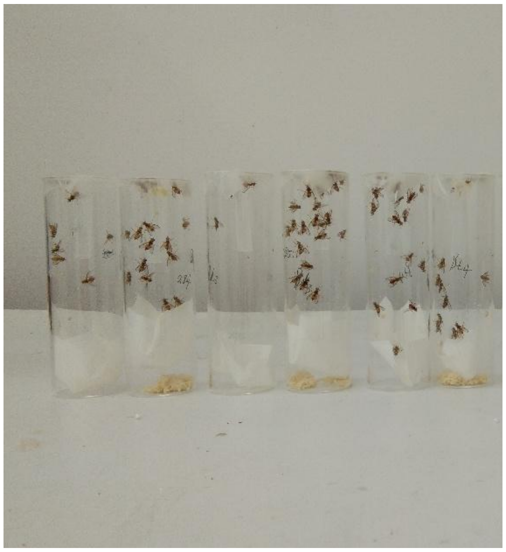

Fabrication of ovipositor

A certain amount of eggs was weighed and placed at the bottom of flat-bottom glass tubes (Φ2.5 cm, h 9 cm). Two pieces of perforated white filter paper (3 cm×3 cm) were placed above the eggs and at 2.0–2.5 cm from the tube orifices, respectively. After adding 600 μL of sterile water, the glass tubes were wrapped with white paper.

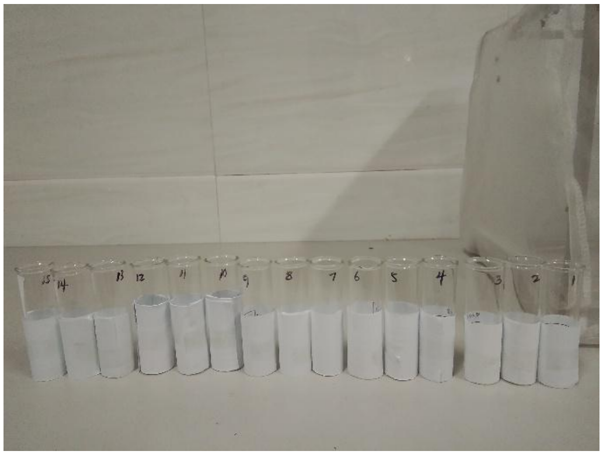

Supplement: Supplementary file 1 [file insects-17-00266-s001.zip › File S1.pdf]
