# Supplementary material for: Volatile Compounds from Eggs of Three Fruit Fly Drive Aggregation and Oviposition
Source: Insects. 2026 Mar 2;17(3):266. doi: 10.3390/insects17030266 (PMC13027054; doi:10.3390/insects17030266)
Supplement: Supplementary file 1 [file insects-17-00266-s001.zip › Table S1.pdf]

Table S1. Volatile compounds and relative contents in eggs of *Bactrocera dorsalis*, *Zeugodacus cucurbitae*, and *Zeugodacus tau* (%).

Number	Chemical compound	Molecular formula	Retention time (min)	*B. dorsalis*	*Z. cucurbitae*	*Z. tau*	Pumpkin
1	1,4-Pentadiene	C_5H_8	1.76	0.04±0.01	—	—	—
2	Heptane	C_7H_{16}	1.88	0.01±0.01	0.49±0.05	1.05±0.08	—
3	2-Methyl-heptane	C_8H_{18}	2.08	—	0.69±0.06	0.24±0.15	—
4	Methyl acetate	$C_3H_6O_2$	2.45	—	3.66±0.64	—	—
5	(+-)-3-Methyl-1-penten-3-ol	$C_6H_{12}O$	2.94	0.18±0.04	2.18±0.13	1.78±0.51	0.18±0.13
6	Unknown 1		2.99	—	2.43±0.16	1.64±0.43	—
7	4-Penten-2-ol	$C_5H_{10}O_2$	3.63	0.04±0.01	1.28±0.18	0.86±0.16	0.25±0.02
8	2,5-Dimethylnonane	$C_{11}H_{24}$	4.13	0.27±0.06	1.53±0.10	1.51±0.39	0.41±0.15
9	5H-naphtho[2,3-c]carbazole	$C_{20}H_{13}N$	4.61	0.00±0.00	0.02±0.01	—	—
10	2,3,4-Trimethylheptane	$C_{10}H_{22}$	5.22	0.10±0.02	0.10±0.10	0.21±0.14	—
11	Amylene hydrate	$C_5H_{12}O$	5.47	—	1.61±0.12	—	—
12	Ethyl isovalerate	$C_7H_{14}O_2$	6.85	0.62±0.09	—	0.04±0.04	—
13	Unknown 2		7.12	0.05±0.02	0.51±0.13	0.40±0.17	—
14	6-Ethyl-2-methyloctane	$C_{11}H_{24}$	7.13	0.62±0.12	3.53±0.19	3.04±0.15	—
15	2,6-Dimethyl-6-trifluoroacetoxyoctane	$C_{12}H_{21}F_3O_2$	7.72	0.24±0.03	0.71±0.18	0.95±0.09	0.72±0.09
16	2-Ethyl-1-dodecanol	$C_{14}H_{30}O$	7.83	0.02±0.00	0.04±0.02	0.02±0.02	0.05±0.01
17	Isobutanol	$C_4H_{10}O$	8.12	0.12±0.05	—	—	—
18	Isoamyl acetate	$C_7H_{14}O_2$	8.33	1.13±0.26	—	—	—
19	3-Methyl-2-butanol	$C_5H_{12}O$	8.69	—	1.43±0.38	0.38±0.25	—
20	7-methyl-7H-Dibenzo[b,g]carbazole	$C_{21}H_{15}N$	9.61	0.01±0.00	0.05±0.03	0.08±0.05	—
21	3-Methylundecane	$C_{12}H_{26}$	9.61	0.01±0.01	0.04±0.04	0.05±0.05	—
22	2,3,5-Trimethyldecane	$C_{13}H_{28}$	9.78	0.06±0.01	0.35±0.14	0.16±0.11	—
23	Unknown 3		9.88	0.06±0.02	0.38±0.16	0.27±0.12	—
24	Isobutyl 2-methylbutyrate	$C_9H_{18}O_2$	9.98	0.06±0.02	—	—	—
25	Cyclopentanone	C_5H_8O	10.07	—	0.54±0.14	—	—
26	2-Methylene-1-pentyl-1-trimethylsilyl-cyclopropane	$C_{12}H_{24}Si$	10.16	0.05±0.03	1.67±0.14	—	—
27	Ethyl aminomethylformimidate	$C_4H_{10}N_2O$	10.17	0.05±0.03	—	0.61±0.38	20.26±7.73
28	Unknown 4		10.36	0.03±0.01	—	—	—
29	Isoamyl propionate	$C_8H_{16}O_2$	10.39	0.12±0.07	—	—	—
30	Isobutyl isovalerate	$C_9H_{18}O_2$	10.44	0.61±0.19	—	—	—
31	Isopentyl isobutyrate	$C_9H_{18}O_2$	10.54	3.15±0.94	—	—	—
32	3-Methylheptan-2-one	$C_8H_{16}O$	10.70	0.04±0.02	0.22±0.09	0.16±0.10	—
33	3,5-Dimethyl-2-octanone	$C_{10}H_{20}O$	10.59	0.09±0.05	0.18±0.08	0.22±0.06	—
34	Methanesulfonic anhydride	$C_2H_6O_5S_2$	10.75	0.27±0.07	2.12±0.56	1.22±0.75	—
35	Unknown 5		10.79	0.16±0.09	—	—	—
36	Cyclobutanecarbonitrile	C_5H_7N	11.17	0.61±0.26	—	—	—
37	4,6-Dimethyldodecane	$C_{14}H_{30}$	11.17	0.18±0.03	1.14±0.07	0.96±0.07	0.34±0.12
38	3,5-Diamino-1,2,4-triazole	$C_2H_5N_5$	11.62	—	0.10±0.04	0.08±0.05	—
39	3-Butyl-2-hydroxy-2-cyclopenten-1-one	$C_9H_{14}O_2$	11.77	0.06±0.01	—	—	—

Number	Chemical compound	Molecular formula	Retention time (min)	*B. dorsalis*	*Z. cucurbitae*	*Z. tau*	Pumpkin
40	(Octadecyl E)-2-methylbut-2-enoate	$C_{23}H_{44}O_2$	12.21	0.08±0.03	—	—	—
41	(o-Methoxy-benzoyl)-tris(trimethylsilyl)-silane	$C_{17}H_{34}O_2Si_4$	12.39	0.02±0.01	0.20±0.02	0.17±0.05	—
42	Unknown 6		12.67	0.15±0.03	—	—	—
43	3-Methylbutyl 2-methylbutanoate	$C_{10}H_{20}O_2$	13.03	2.81±0.80	—	—	—
44	Octyl cyclopentanecarboxylate	$C_{14}H_{26}O_2$	13.47	26.81±5.42	15.00±4.73	7.77±0.68	—
45	Desmethyldeprenyl	$C_{12}H_{15}N$	13.46	0.01±0.00	—	—	—
46	Isoamyl isovalerate	$C_{10}H_{20}O_2$	13.56	1.83±0.48	—	—	—
47	Unknown 7		13.66	0.31±0.13	—	—	—
48	Tetracosyl cyclopentanecarboxylate	$C_{30}H_{58}O_2$	14.20	0.15±0.07	0.03±0.03	0.05±0.05	—
49	Eicosyl octyl ether	$C_{28}H_{58}O$	14.57	0.33±0.07	2.51±0.20	1.97±0.18	0.18±0.09
50	1-Chlorooctadecane	$C_{18}H_{37}Cl$	14.71	0.02±0.01	0.13±0.01	0.07±0.04	0.30±0.21
51	Anisole	C_7H_8O	14.67	0.31±0.11	—	—	—
52	11αHydroxy-tetrahydro-solasodine	$C_{27}H_{47}NO_3$	14.85	0.02±0.01	—	—	—
53	Glutaric acid, but-3-yn-2-yl 8-chlorooctyl ester	$C_{17}H_{27}ClO_4$	14.89	0.02±0.01	—	—	—
54	2-Hydroxy-3-pentanone	$C_5H_{10}O_2$	15.18	0.16±0.05	—	—	0.48±0.34
55	Dodecyl cyclopentanecarboxylate	$C_{18}H_{34}O_2$	15.71	2.75±0.54	—	0.06±0.04	—
56	Unknown 8		15.79	0.45±0.13	—	—	—
57	Unknown 9		15.88	0.03±0.02	0.38±0.02	0.27±0.07	—
58	Unknown 10		16.82	0.02±0.00	0.15±0.01	0.14±0.05	—
59	3,7,11,15-tetramethylhexadecan-1,2,3-triol silylated	$C_{29}H_{66}O_3Si_3$	16.94	—	0.19±0.08	0.20±0.13	—
60	1-Hydroxycyclohexanecarboxylic acid	$C_7H_{12}O_3$	17.01	—	—	0.32±0.03	—
61	2-Methyl-3-hexanol	$C_7H_{16}O$	17.23	0.09±0.03	—	—	4.24±0.62
62	2-[2-(2-acetyloxyethoxy)phenoxy]ethyl acetate	$C_{14}H_{18}O_6$	17.32	0.04±0.01	—	—	—
63	2,3,4-Trimethyl-1-pentanol	$C_8H_{18}O$	17.43	0.01±0.01	0.34±0.09	0.17±0.10	—
64	3-Cyclopentylpropionic acid, 3-pentadecyl ester	$C_{23}H_{44}O_2$	17.53	0.15±0.02	0.26±0.04	0.40±0.05	—
65	1-Tridecene	$C_{13}H_{26}$	17.65	—	0.26±0.07	0.17±0.07	—
66	2-Ethylhexyl cyclopentanecarboxylate	$C_{14}H_{26}O_2$	17.86	0.31±0.05	1.40±0.17	0.52±0.21	—
67	Unknown 11		18.04	0.16±0.04	—	—	—
68	5-Hydroxy-2,7-dimethyl-octan-4-one	$C_{10}H_{20}O_2$	18.23	4.03±0.57	—	—	—
69	1,4-Dichlorobenzene	$C_6H_4Cl_2$	18.49	0.03±0.00	—	0.02±0.02	—
70	2-Ethyl-2-methyl-tridecanol	$C_{16}H_{34}O$	18.92	0.02±0.00	0.11±0.01	0.12±0.02	0.07±0.02
71	Docosane	$C_{22}H_{46}$	19.04	0.33±0.09	2.24±0.15	1.85±0.30	1.28±0.13
72	3-Ethyl-3-methylnonadecane	$C_{22}H_{46}$	19.16	0.06±0.05	0.17±0.12	0.06±0.06	1.50±0.55
73	Heneicosane	$C_{21}H_{44}$	19.30	0.35±0.09	2.38±0.19	2.12±0.34	1.25±0.23

Number	Chemical compound	Molecular formula	Retention time (min)	*B. dorsalis*	*Z. cucurbitae*	*Z. tau*	Pumpkin
74	Fumaric acid, 3,3-dimethylbut-2-yl tridecyl ester	$C_{23}H_{42}O_4$	19.61	0.00±0.00	0.12±0.07	0.16±0.10	—
75	2-Nonanol	$C_9H_{20}O$	19.68	0.02±0.02	3.84±1.31	3.72±0.40	—
76	2,7-dimethyl-4,5-Octanediol	$C_{10}H_{22}O_2$	19.84	0.10±0.05	—	—	—
77	Octadecane	$C_{18}H_{38}$	19.89	0.29±0.08	1.89±0.14	1.98±0.30	3.31±1.65
78	Butanal, 3-methyl-, oxime	$C_5H_{11}NO$	19.94	0.07±0.02	—	—	—
79	3-Oxobutan-2-yl 2-methylbutanoate	$C_9H_{16}O_3$	20.09	0.01±0.01	—	—	—
80	Unknown 12		20.30	0.61±0.13	2.14±0.59	2.46±0.38	—
81	3,5-Dimethyloxolan-2-one	$C_6H_{10}O_2$	20.52	0.01±0.01	1.07±0.08	0.55±0.01	—
82	1-Butoxy-2-methylbutane	$C_9H_{20}O$	20.91	0.23±0.09	1.01±0.43	1.11±0.46	—
83	5,6,7,8-Tetrahydroindolizin	$C_8H_{11}N$	21.23	0.90±0.30	—	—	—
84	Unknown 13		21.35	0.12±0.02	0.03±0.03	—	—
85	Methyl valerate	$C_6H_{12}O_2$	21.41	—	0.03±0.03	0.23±0.07	—
86	6-Methylheptan-2-one	$C_8H_{16}O$	21.47	2.87±0.36	4.77±0.40	11.97±1.37	58.56±6.79
87	(Z)-3-Octen-2-ol	$C_8H_{16}O$	22.06	0.17±0.02	1.01±0.11	1.36±0.09	1.51±0.22
88	1,4,6-Trimethyl-2(1H)-pyridinone	$C_8H_{11}NO$	22.24	0.03±0.01	—	—	—
89	4-O-Acetyl-2,5-di-O-methyl-3,6-dideoxy-d-gluconitrile	$C_{10}H_{17}NO_4$	22.32	—	0.26±0.11	0.43±0.11	—
90	2-Phenylaziridine	C_8H_9N	22.34	0.05±0.02	—	—	—
91	(E)-5-Eicosene	$C_{20}H_{40}$	22.68	—	0.37±0.04	0.35±0.03	—
92	2,6,10,15-Tetramethylheptadecane	$C_{21}H_{44}$	22.75	0.06±0.02	0.45±0.04	0.51±0.09	0.43±0.03
93	Anethole	$C_{10}H_{12}O$	23.02	—	—	0.07±0.02	—
94	Nonadecane	$C_{19}H_{40}$	24.34	0.07±0.02	0.39±0.06	0.56±0.06	—
95	2-Undecanol	$C_{11}H_{24}O$	24.71	—	2.21±0.33	1.81±0.16	—
96	2,6-Diethylcyclohexanone	$C_{10}H_{18}O$	24.72	0.04±0.01	—	—	—
97	2,5-Dimethyltetradecane	$C_{16}H_{34}$	24.79	—	0.39±0.16	0.77±0.22	—
98	Carbonic acid, eicosyl vinyl ester	$C_{23}H_{44}O_3$	24.89	0.04±0.01	0.29±0.05	0.33±0.04	0.23±0.08
99	2-Acetyl-2-thiazoline	C_5H_7NOS	24.93	0.01±0.00	0.26±0.01	0.27±0.02	—
100	(9R,10S)-9,10,16-Trihydroxyhexadecanoic acid	$C_{16}H_{32}O_5$	25.41	0.26±0.05	—	—	—
101	Formamide	CH_3NO	25.51	0.27±0.04	5.66±0.77	3.50±0.41	—
102	Unknown 14		25.63	0.06±0.03	0.23±0.11	0.66±0.10	—
103	N-Acetyl-DL-aspartic acid	$C_6H_9NO_5$	25.66	—	1.26±0.07	1.64±0.30	—
104	2-Methylheptanoic acid	$C_8H_{16}O_2$	25.80	0.08±0.01	0.24±0.06	0.05±0.05	—
105	Carbonic acid, octadecyl vinyl ester	$C_{21}H_{40}O_3$	25.90	0.01±0.01	—	0.36±0.10	—
106	Unknown 15		26.14	0.08±0.02	0.20±0.02	0.39±0.05	—
107	2-Tridecanone	$C_{13}H_{26}O$	26.27	0.55±0.08	1.04±0.06	2.84±0.33	4.25±0.62
108	(1R,2R,4S)-2-(6-Chloropyridin-3-yl)-7-methyl-7-azabicyclo[2.2.1]heptane	$C_{12}H_{15}ClN_2$	26.47	0.02±0.01	—	0.13±0.03	—
109	(Z)-3-Decenyl acetate	$C_{12}H_{22}O_2$	26.61	0.51±0.09	0.43±0.04	1.50±0.22	—
110	Unknown 16		26.99	—	0.00±0.00	0.01±0.00	—
111	Ethyl dodecanoate	$C_{14}H_{28}O_2$	27.04	17.62±6.58	1.29±0.09	1.96±0.22	—
112	(Z)-14-Tricosenyl formate	$C_{24}H_{46}O_2$	27.16	0.09±0.03	—	—	—

Number	Chemical compound	Molecular formula	Retention time (min)	*B. dorsalis*	*Z. cucurbitae*	*Z. tau*	Pumpkin
113	Isoamyl decanoate	$C_{15}H_{30}O_2$	27.45	0.02±0.00	—	—	—
114	N-Hexyl-propanamide	$C_9H_{19}NO$	27.70	—	1.96±0.27	0.27±0.02	—
115	N-(3-Methyl-2-butenyl)acetamide	$C_7H_{13}NO$	27.95	0.00±0.00	0.55±0.06	0.30±0.03	—
116	Methyl 15-methylhexadecanoate	$C_{18}H_{36}O_2$	28.00	0.09±0.03	—	—	—
117	Perhydropyrene	$C_{16}H_{26}$	28.27	—	0.30±0.05	0.46±0.04	—
118	Tetracosane	$C_{24}H_{50}$	28.27	0.03±0.02	—	—	—
119	Ethyl tridecanoate	$C_{15}H_{30}O_2$	28.48	0.13±0.03	—	—	—
120	Propyl dodecanoate	$C_{15}H_{30}O_2$	28.82	0.17±0.08	—	—	—
121	1-Allyloxy-1-ethyl-1-silacyclohexane	$C_{10}H_{20}O$ Si	29.30	0.02±0.01	—	—	—
122	Isobutyl laurate	$C_{16}H_{32}O_2$	29.45	0.03±0.01	—	—	—
123	2-Ethylhexanoic acid	$C_8H_{16}O_2$	29.60	0.12±0.06	—	—	—
124	N-tert-butyldimethylsily-propargylamine	$C_9H_{19}NSi$	29.62	0.24±0.04	0.30±0.08	0.44±0.05	0.20±0.14
125	6-(3-Hydroxy-4-methylphenyl)-2-methylhept-2-en-4-one	$C_{15}H_{20}O_2$	29.68	—	0.28±0.09	0.49±0.04	—
126	Biphenyl	$C_{12}H_{10}$	29.77	0.01±0.00	—	0.07±0.03	—
127	3,3,6-trimethyl-Bicyclo[3.1.0]hexan-2-one,	$C_9H_{14}O_2$	30.18	—	0.31±0.04	0.10±0.06	—
128	Methyleugenol	$C_{11}H_{14}O_2$	30.34	0.01±0.00	—	6.84±1.20	—
129	Guanidine carbonate	$C_3H_{12}N_6O_3$	30.35	—	0.44±0.02	—	—
130	Methyl tetradecanoate	$C_{15}H_{30}O_2$	30.42	0.13±0.04	0.25±0.02	0.50±0.06	—
131	2-Methoxy-4,5,6-trimethylpyrimidine	$C_8H_{12}N_2O$	30.56	0.03±0.00	0.15±0.02	—	—
132	3-Amino-5-fluoropyridine	$C_5H_5FN_2$	30.88	0.05±0.02	—	—	—
133	1-Isopropyl-2,2-dimethylpropylideneamine	$C_8H_{17}N$	30.90	—	0.60±0.03	0.74±0.13	—
134	Ethyl tetradecanoate	$C_{16}H_{32}O_2$	31.21	14.01±3.07	4.01±0.21	6.32±0.95	—
135	Unknown 17		31.42	0.03±0.01	—	—	—
136	Isoamyl laurate	$C_{17}H_{34}O_2$	31.56	1.19±0.15	—	—	—
137	Ethyl 3-(2,6-dimehylmorpholino)propionate	$C_{11}H_{21}NO_3$	31.63	0.10±0.01	—	—	—
138	Ethyl 9-tetradecenoate	$C_{16}H_{30}O_2$	31.65	0.38±0.11	—	—	—
139	Isopentyl undec-10-enoate	$C_{16}H_{30}O_2$	31.81	0.01±0.00	—	—	—
140	Unknown 18		31.83	—	0.11±0.03	0.09±0.06	—
141	Unknown 19		31.91	0.18±0.04	0.23±0.02	0.17±0.03	—
142	Methyl 3-(methylthio)propionate	$C_5H_{10}O_2S$	32.06	—	—	1.43±0.43	—
143	[R-(Z)]-9-Octadecenoic acid, 12-hydroxy-, ethyl ester	$C_{20}H_{38}O_3$	32.13	0.01±0.01	—	—	—
144	Methylisoeugenol	$C_{11}H_{14}O_2$	33.52	—	—	0.12±0.01	—
145	4,4-Dimethyl-1,3-dioxane	$C_6H_{12}O_2$	34.27	—	0.09±0.06	0.33±0.14	—
146	Methyl palmitoleate	$C_{17}H_{32}O_2$	34.80	0.03±0.01	0.19±0.01	0.22±0.03	—
147	3-Methylbutyl tetradecanoate	$C_{19}H_{38}O_2$	35.35	0.62±0.08	—	—	—
148	Ethyl 9-hexadecenoate	$C_{18}H_{34}O_2$	35.47	2.57±0.58	5.49±0.42	3.99±0.65	—
149	Glutarimide	$C_5H_7NO_2$	35.93	0.60±0.10	0.92±0.11	0.80±0.08	—

Number	Chemical compound	Molecular formula	Retention time (min)	*B. dorsalis*	*Z. cucurbitae*	*Z. tau*	Pumpkin
150	cis-9-Tetradecenoic acid, heptyl ester	$C_{21}H_{40}O_2$	36.01	0.06±0.02	—	—	—
151	Unknown 20		36.87	0.05±0.01	—	—	—
152	1,3-Dimethyluracil	$C_6H_8N_2O_2$	38.48	0.05±0.01	—	—	—
153	Ethyl arachidate	$C_{22}H_{44}O_2$	38.55	0.04±0.01	0.15±0.04	—	—
154	Unknown 21		38.84	0.46±0.03	—	—	—
155	Pentyl palmitoleate	$C_{21}H_{40}O_2$	39.26	1.22±0.16	—	—	—
156	Lauric acid	$C_{12}H_{24}O_2$	39.41	0.59±0.24	—	0.79±0.35	—
157	Amyl elaidate	$C_{23}H_{44}O_2$	42.36	0.04±0.02	—	—	—
158	Myristic acid	$C_{14}H_{28}O_2$	42.79	0.08±0.05	—	0.37±0.17	—
159	Palmitoleic acid	$C_{16}H_{30}O_2$	46.55	0.48±0.19	—	1.05±0.65	—

Values are expressed as mean percentage of total volatiles ± standard error.
